# Altered Topological Properties of White‐Matter Functional Networks in Young Smokers

**DOI:** 10.1111/adb.70125

**Published:** 2026-02-24

**Authors:** Zhenzhen Mai, Dahua Yu, Gengdi Huang, Xiaojiao Li, Xuwen Wang, Fang Dong, Yongxin Cheng, Juan Wang, Yuxin Ma, Lin Luo, Kai Yuan, Ting Xue

**Affiliations:** ^1^ School of Digital and Intelligent Industry Inner Mongolia University of Science and Technology Baotou Inner Mongolia China; ^2^ School of Automation and Electrical Engineering Inner Mongolia University of Science and Technology Baotou Inner Mongolia China; ^3^ Department of Addiction Medicine Shenzhen Kangning Hospital, Shenzhen Mental Health Center Shenzhen China; ^4^ State Key Laboratory of Chemical Oncogenomics, Guangdong Provincial Key Laboratory of Chemical Genomics Peking University Shenzhen Graduate School Shenzhen China; ^5^ Imaging Department Medical Science of the First Affiliated Hospital of Baotou Medical College Baotou Inner Mongolia China; ^6^ Life Sciences Research Center, School of Life Science and Technology Xidian University Xi'an Shaanxi China; ^7^ Hainan Free Trade Port Health Medical Research Institute Baoting Hainan China; ^8^ School of Science College Inner Mongolia University of Science and Technology Baotou Inner Mongolia China

**Keywords:** fMRI, functional network, graph theory, white matter, young smokers

## Abstract

Smoking addiction is a common mental disorder, and numerous imaging studies have shown that adolescents with smoking addiction exhibit abnormalities in brain function and structure. This study aims to investigate changes in the topological characteristics of the white matter (WM) functional network in young smokers. Forty‐two young smokers and 42 age‐, gender‐, and education‐matched nonsmokers were included in the study. The functional connectome of white matter and graph theory were used to study these participants. Two‐sample *t*‐test were used for the detection of the abnormal graph properties in young smokers. Pearson correlation was applied for the correlation analyses between properties and clinical indicators of smoking. The global level WM functional network analyses showed that the $C_{p}$ and $E_{\text{local}}$ values were higher in young smokers than in the control group, and the $E_{\text{local}}$ was positively correlated with the age of first smoking. At the node level, five nodes of the WM functional network exhibited abnormal node properties in the WM regions of the bilateral hippocampal parahippocampal gyrus (CGH), bilateral superior longitudinal fasciculus (SLF), bilateral inferior frontal gyrus (IFO), middle cerebellar peduncle (MCP), and the bilateral anterior commissure (ACR) in young smokers. The node degree centrality value of MCP was positively correlated with age of first smoking. Our neuroimaging findings provide evidence of WM functional alterations associated with nicotine addiction, which may enhance our understanding of the neural mechanisms underlying smoking addiction in young smokers.

## Graphical abstract

The functional connectome of white matter and graph theory were used to study these participants. The global level WM functional network analyses showed that the $C_{p}$ and $E_{\text{local}}$ values were higher in young smokers than in the control group, and the $E_{\text{local}}$ was positively correlated with the age of first smoking. At the node level, five nodes of the WM functional network exhibited abnormal node properties in the WM regions.

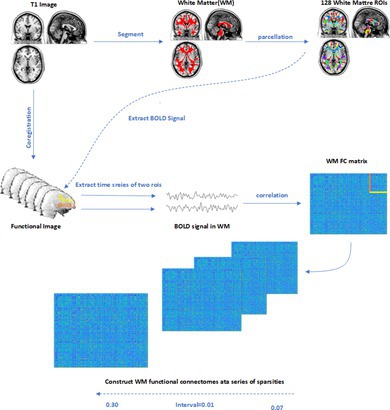

## 1 Introduction

Cigarettes are the most destructive to national public health (C et al.). Almost 100% of smoking initiation occurs during childhood and adolescence (AM and MP). Tobacco use during adolescence is closely related to later tobacco use [1]. Research focused on the neurobiological mechanisms of nicotine dependence in young smokers may enhance understanding of the pathogenesis of nicotine addiction and thereby inform the development of effective intervention strategies.

Long‐term smoking may lead to a wide range of structural and functional brain damage [2]. Neuroimaging evidence has indicated that drug craving is closely linked to functional connectivity within the frontoparietal network [3]. Previous studies have detected significant changes in cortical thickness or grey matter density in the anterior cingulate cortex (ACC), prefrontal cortex (PFC), and striatal volumes in young smokers [4, 5]. Additionally, gaming addiction and smartphone addiction are prevalent among young people. Researchers have identified similarities in the implications of frontostriatal circuits between internet gaming disorder (IGD) and nicotine addiction, such as grey matter volume/density, white matter properties, and resting state abnormalities, possibly qualifying IGD as a true addiction especially in light of the current social situations. Both have a top‐down approach of the cortical regions to the striatum with overlapping reward and cognitive control pathways that play a crucial role in modulating addictive behaviour [6]. White matter damage is a core pathological defect in the brains of smoking addicts [7], and it has been shown that exposure to tobacco smoke during adolescence disrupts the white matter microstructures, particularly in the prefrontal cortex regions and the internal capsule [8]. For example, prior studies have revealed white matter (WM) morphological deficits or microstructural integrity, such as the corpus callosum, cingulum, and frontoparietal fibre tracts, in cases of nicotine addiction [9, 10, 11]. It is worth noting that most existing studies focus on white matter structural changes, and few studies focus on its functional changes; while a large number of recent studies have shown that the functional signals of brain white matter have certain physiological significance [12, 13, 14, 15]. Since white matter provides information transmission within the brain, enabling the rapid and efficient integrative capacity of neural systems necessary for the highly evolved cognitive operations of mankind [16]; clarifying the changes in white matter function of young smokers tobacco use addicts is essential for understanding tobacco use addiction.

Blood oxygen level‐dependent (BOLD)‐based functional magnetic resonance imaging (fMRI) functional networks are usually computed by temporal correlation of BOLD‐fMRI signals between distributed brain regions, which are thought to reflect neural activities or related functions occurring in grey matter (GM) [17]. An increasing number of studies in recent years have utilized BOLD‐fMRI to detect functional brain activity in the WM. For example, it has been found that different subregions of the corpus callosum can be exclusively activated by their functionally relevant tasks [18, 19]. Low‐frequency BOLD fluctuations in specific WM bundles can be modulated by different tasks [15, 20, 21]. The intensity of WM functional brain activity was found to be closely related to participants' performance on cognitive tasks [22]. Moreover, specific WM tracts, identified from BOLD‐fMRI signals, showed a similar pattern with the fibre bundles from DTI tracking in the human brain [15, 23]. All these evidences demonstrate that white matter fMRI signals contain information about brain functional activities, rather than just considering white matter signals as noise or artefacts [15, 22].

Recent studies have also found that WM_BOLD signals in the resting state show topological organization rather than random distribution like noise [12, 13, 14, 15]. It has been demonstrated that the white matter functional networks have small‐world properties [24]; furthermore, the WM functional network changes have been identified in individuals with schizophrenia, benign epilepsy with centrotemporal spikes, depression, and Parkinson's disease, suggesting a potential neuropathological process leading to brain dysfunction [17, 25, 26, 27, 28]. Abnormalities in white matter functional networks have also been detected in adolescent smoking addicts [29, 30]. Given that smoking addiction causes diffuse brain damage to the brain, this may lead to abnormal changes in the topological properties of white matter functional networks. Therefore, in the present study, we used rs‐fMRI combined with graph theoretical analysis to explore the characteristic changes of functional WM network topological properties in adolescent smokers. The correlation analyses were conducted between the altered topological parameters and related clinical variables. We hoped that the current study focused on topological properties of the WM network may improve our understanding of nicotine addiction.

## 2 Methods

### 2.1 Study Subjects

This study was approved by the Medical Ethics Committee of the First Affiliated Hospital of Baotou Medical College, Inner Mongolia University of Science and Technology (approval number: 20200326). All experimental procedures were implemented in strict accordance with the latest guidelines for human medical research, the Declaration of Helsinki of the World Medical Association. The participants of the experimental data collection were fully informed about all the experimental procedures and related precautions before the start of the experiment, and their written informed consent was obtained.

The young smokers and healthy controls were recruited from the First Affiliated Hospital of Baotou Medical College of Inner Mongolia University of Science and Technology. All subjects did not have physical illness (brain tumour, obstructive lung disease, hepatitis, or epilepsy), neurological disease (e.g., stroke), or claustrophobia according to clinical evaluations and medical records. None of the subjects reported daily consumption of alcohol, drug abuse or dependence (other than nicotine dependence for young smokers), or medications using currently that may affect cognitive functioning. Nicotine dependence levels in young smokers were assessed with the FTND [31]. For young smokers who meet the inclusion criteria, the FTND assessment score is ≥ 2 points [32]. Young male smokers were selected based on the diagnostic criteria for nicotine dependence in the Diagnostic and Statistical Manual of Mental Disorders, Fifth Edition (DSM‐5). The main inclusion criteria were: smoking behaviour lasting for more than 2 years; the number of cigarettes smoked per day being ≥ 10; and no smoking cessation attempts in the past 6 months. Healthy male non‐smokers, matched for age and education level, were recruited from non‐smoking dormitories. Neither of their parents smoked to avoid the effects of secondhand smoke exposure. The pack‐year of smoking means that someone had smoked one package of cigarettes (20 cigarettes) daily for 1 year, which characterizes the cumulative amount of nicotine intake. CO level in expired air measured by the Smokerlyzer System (Bedfont Scientific Ltd., Rochester, UK) was verified as 10 ppm in young smokers and 3 ppm in nonsmokers [33]. All of the participants were male right‐handed [34], with no significant differences in age or years of education. A total of 48 young smokers and 48 healthy controls were screened in this study.

### 2.2 MRI Data Acquisition

After completing all questionnaires, the subjects were scanned with magnetic resonance imaging (MRI). The experiments were performed in a 3 T Philips MRI scanner at the First Affiliated Hospital of Baotou Medical College. During scanning, the heads of all participants were fixed with a foam pad to reduce head movement. Ear plugs were used to minimize noise. Resting‐state fMRI data were acquired using a gradient echo‐echo planar imaging sequence with the following scanning parameters: repetition time 2 s; echo time 30 ms; matrix, 64 × 64; field of view, 220 mm; flip angle 90°; scanning time, 6 min. During the scanning process, all participants were required to keep their entire body still and eyes closed.

### 2.3 Brain Imaging Data Preprocessing

In this study, functional images were preprocessed by using DPABI (http://www.restfmri.net) and SPM12 (http://www.fil.ion.ucl.ac.uk/spm/software/spm12). For further analysis, the DICOM images were transformed into Nifti format files using the dcm2niix tool. The first 10 volumes of fMRI scans were removed. Next, slice‐timing correction and realignment were performed. T1 anatomical images were then coregistered to the functional images and further segmented into tissue probability maps of white matter, grey matter, and cerebrospinal fluid (CSF) by using a diffeomorphic nonlinear registration algorithm in SPM12.

To minimize the partial‐volume effect of GM signals, only WM functional images were preprocessed for subsequent analysis. The probability map of WM (a rigorous 90% threshold) produced by structural segmentation was used to generate WM individual mask [13, 23, 24]. Each participant's WM functional images were spatially extracted from WM individual masks by using the dot product [26, 27, 28]. WM functional images were then normalized to Montreal Neurological Institute (MNI) space and resampled into 3 × 3 × 3 mm^3^. To produce a group‐level WM mask, only voxels labelled as WM across 80% of the participants were included [23, 24].

To avoid the mixing effect of deep brain structures, the Harvard‐Oxford Atlas (25% probability) was used to remove subcortical nuclei (i.e., the bilateral thalamus, putamen, caudate, pallidum, and nucleus accumbens) from the group‐level WM mask [35]. In case of generating spurious local spatial correlation between voxels, spatial smoothing was not performed [14, 26]. Bandpass filtering (0.01–0.10 Hz) was also applied to minimize nonneuronal sources. Scrubbing parameters were used to regress out any confounding effects; specifically, we removed the signal value at a point with a framewise displacement > 0.5 mm, along with the signal values of one forward neighbour and two back neighbours [14, 26].

### 2.4 WM Functional Connectome Construction

**FIGURE 1:**
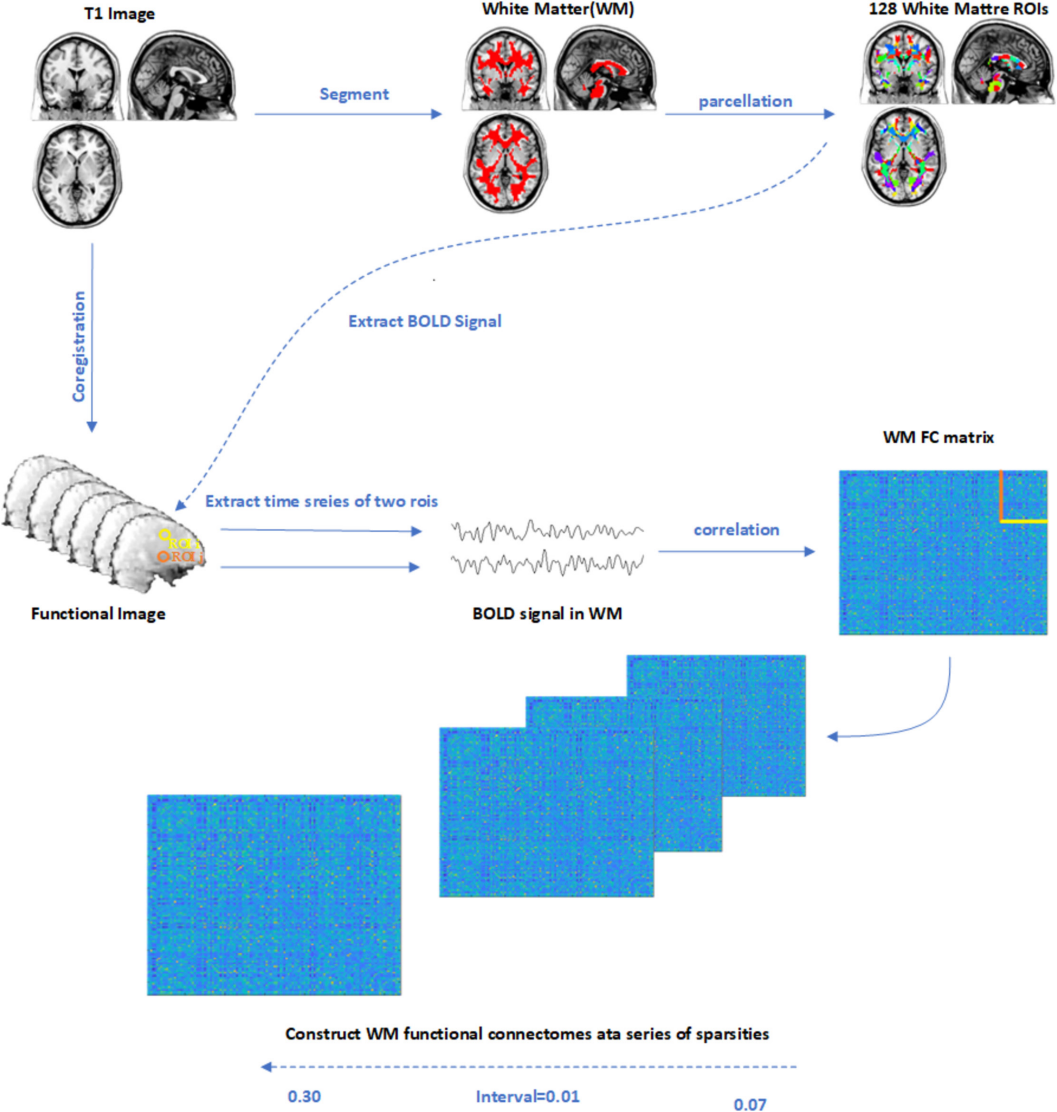
Flow chart of WM functional connectivity construction: (I) The structural images were coregistered with preprocessed functional images. (II) The coregistered structural images were segmented into WM, GM, and CSF, generating a group‐level WM mask. (III) The group‐level WM mask was randomly separated into 128 anatomical nodes of approximately equal size. (IV) BOLD signals of WM were obtained, and the time series of each node was extracted. (V) WM FC matrices were computed by using Pearson's correlation between each pair of nodes. (VI) WM functional connectivity were constructed at a series of sparsity from 0.07 to 0.30 (interval = 0.01).

To define nodes in WM functional connectomes [24, 25], the group‐level WM mask was randomly subdivided into N (here, *N* = 128) contiguous anatomical regions while constraining the size of nodes as uniformly as possible using a region‐growing method and marked using JHU‐Atlas [28, 36]. As previously described by previous studies [36], N seed voxels in WM were randomly chosen, each of which corresponds to the first voxel to be classified as belonging to each of the N nodes. All other voxels in WM remain unlabeled. The strategy was to incrementally ‘grow’ each node voxel‐by‐voxel until every WM voxel has been assigned to exactly one node. At each iteration of the growth phase, a new voxel is assigned to the node with the smallest volume [36]. Finally, each subject's correlation matrix (128 × 128) was constructed by Pearson's correlation coefficient between averaged time series within each paired node. Then, Fisher r to Z transformation was applied to the correlation matrices. Topological properties were evaluated based on the weighted WM functional connectomes. The flowchart was shown in Figure 1.

### 2.5 Network Properties of WM Functional Connectomes

To mitigate the influence of thresholding on network topological properties, this study employs a sparsity‐based threshold selection method to binarize the correlation matrix and utilizes graph theory to characterize the topological features of WM functional connectomes across various sparsity thresholds [37]. The sparsity of a network can be considered as the ratio of the total number of edges to the maximum number of edges possible. The range of sparsity should satisfy the following condition: First, the average degree (the degree of a node is the number of connections linked to the node) over all nodes of each thresholded network was larger than 2 × log(N), with *N* = 128 here denoting the number of nodes. Second, all thresholds of small‐worldness (σ) were larger than 1.0 among all participants [26, 38, 39]. Based on this, we acquired a range for the sparsity threshold of the WM functional network (0.07–0.30, with an interval of 0.01). Global and nodal topological properties were acquired by Gretna (www.nitrc.org/projects/gretna) based on the MATLAB R2017b platform. The following global parameters were included: normalized clustering coefficient (γ), normalized shortest path length (λ), small‐worldness (σ), clustering coefficient ($C_{P}$), shortest path length ($L_{p}$), global efficiency ($E_{\text{global}}$), local efficiency ($E_{\text{local}}$), assortativity (α), synchronization (S), and hierarchy (β). Meanwhile, the following nodal parameters were included in the study: betweenness centrality (bc), degree centrality (DC), node clustering coefficient ($NC_{p}$), node efficiency (Ne), node shortest path length ($NL_{p}$), and node local efficiency ($NL_{e}$).

A review outlined the uses and interpretations of these topological properties [40]. $E_{\text{local}}$ measures the efficiency of information transmission within the neighbourhood of each node in the network, reflecting the fault tolerance and information redundancy of local subnetworks. $E_{\text{global}}$ quantifies the overall efficiency of global information transfer. $C_{p}$ measures the degree of nodes tending to cluster together. $L_{p}$ reflects the efficiency of global information transmission. λ reflects the relative degree of local node aggregation. γ reflects the relative efficiency of global information transfer. σ indicates whether the network exhibits “high clustering, short path” small‐world characteristics. α refers to the tendency of nodes to connect with other nodes of similar degree, and it indicates the assortative (or disassortative) nature of node connections. S represents the degree of coordinated dynamic behaviour among network nodes. β describes the extent of hierarchical organization in the network's modular structure. $NL_{e}$ reflects the fault tolerance of a network, indicating the communication status between the given node and its neighbourhood when the node is deleted. $NL_{p}$ measures the likelihood of a node being connected to its neighbouring node. Ne reflects the node's efficiency in global information transfer. $NC_{p}$ quantifies the cohesion of the local subnetwork around that node. bc reflects a node's hub role in global information transfer. DC measures the local connection strength of a node. We also calculated the area under the curve (AUC) for each topological property. The AUC provides a summarized scalar for topological properties of the WM functional connectome and provides a sensitive way to detect topological abnormalities in brain disorders [39].

### 2.6 Statistical Analyses

This study used GraphPad Prism 9 (www.graphpad.com) for statistical analysis of demographic and neuropsychological data. Group differences in age and education were evaluated using two‐sample *t* tests. The statistical analysis of the topological properties was carried out using SPSS 22 (https://www.ibm.com/cn‐zh/products/spss). The differences of each sparsity and those of the AUC (area under curve) were tested by two‐sample *t*‐test under the model of general linear model. The differences of the nodal properties were tested only on the AUC condition by using the same method of the global properties. The significance threshold was set at *p* < 0.05, and Bonferroni correction was used for multiple comparison correction. Pearson and Spearman correlation analyses were applied to examine the correlations between clinical variables and network properties.

## 3 Results

### 3.1 Demographic and Clinical Characteristics

Six young smokers and six healthy controls were excluded due to failure to complete all T1 and resting‐state fMRI scans, or head motion exceeding 2 mm or 2°. The remaining 42 young smokers and 42 healthy controls were matched for age (*p* = 0.623) and education level (*p* = 0.123). The demographic and clinical characteristics of these participants are shown in Table 1.

**TABLE 1:** Participant demographic and clinical characteristics.

	Young smokers (*n* = 42)	Controls (*n* = 42)	*p*
Age (years)	19.50 ± 2.12	19.55 ± 1.86	0.95
Sex (M/F)	42/0	42/0	—
Duration of smoking	4.40 ± 2.96	—	—
FTND	6.95 ± 1.97	—	—
Cigarettes per day (CPD)	16.23 ± 5.60	—	—
Age of first smoking	14.23 ± 2.57	—	—
Pack‐years	3.57 ± 2.84	—	—
Strong hand	Dextral	Dextral	—
Educational level	Bachelor's degree	Bachelor's degree	—

*Note:* Data are presented as mean ± standard deviation. Pack‐years: smoking years × daily consumption/20.

Abbreviation: FTND: Fagerström Test for Nicotine Dependence.

### 3.2 Group Differences of Global Properties

**FIGURE 2:**
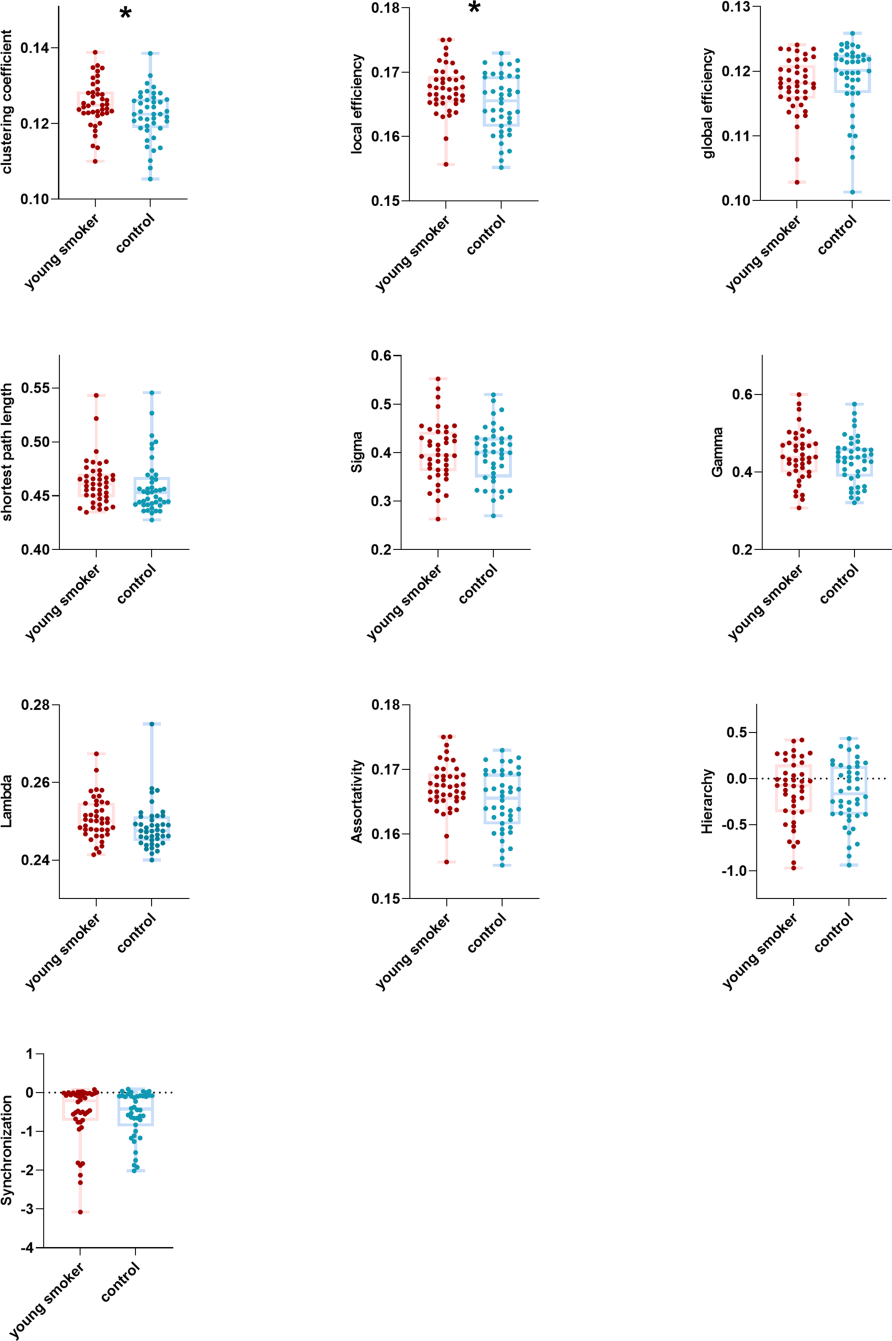
Statistical analyses for the global properties between young smokers and healthy controls (HCs). Between‐group comparisons of the AUC values showed increased $C_{p}$ (*p* = 0.034*), and $E_{\text{local}}$ (*p* = 0.017*) in young smokers. No differences were observed in the λ(*p* = 0.090), σ (*p* = 0.639), γ(*p* = 0.414), $L_{p}$ (*p* = 0.484), $E_{\text{global}}$ (*p* = 0.313), β (*p* = 0.752), α (*p* = 0.133), and S (*p* = 0.944).

**FIGURE 3:**
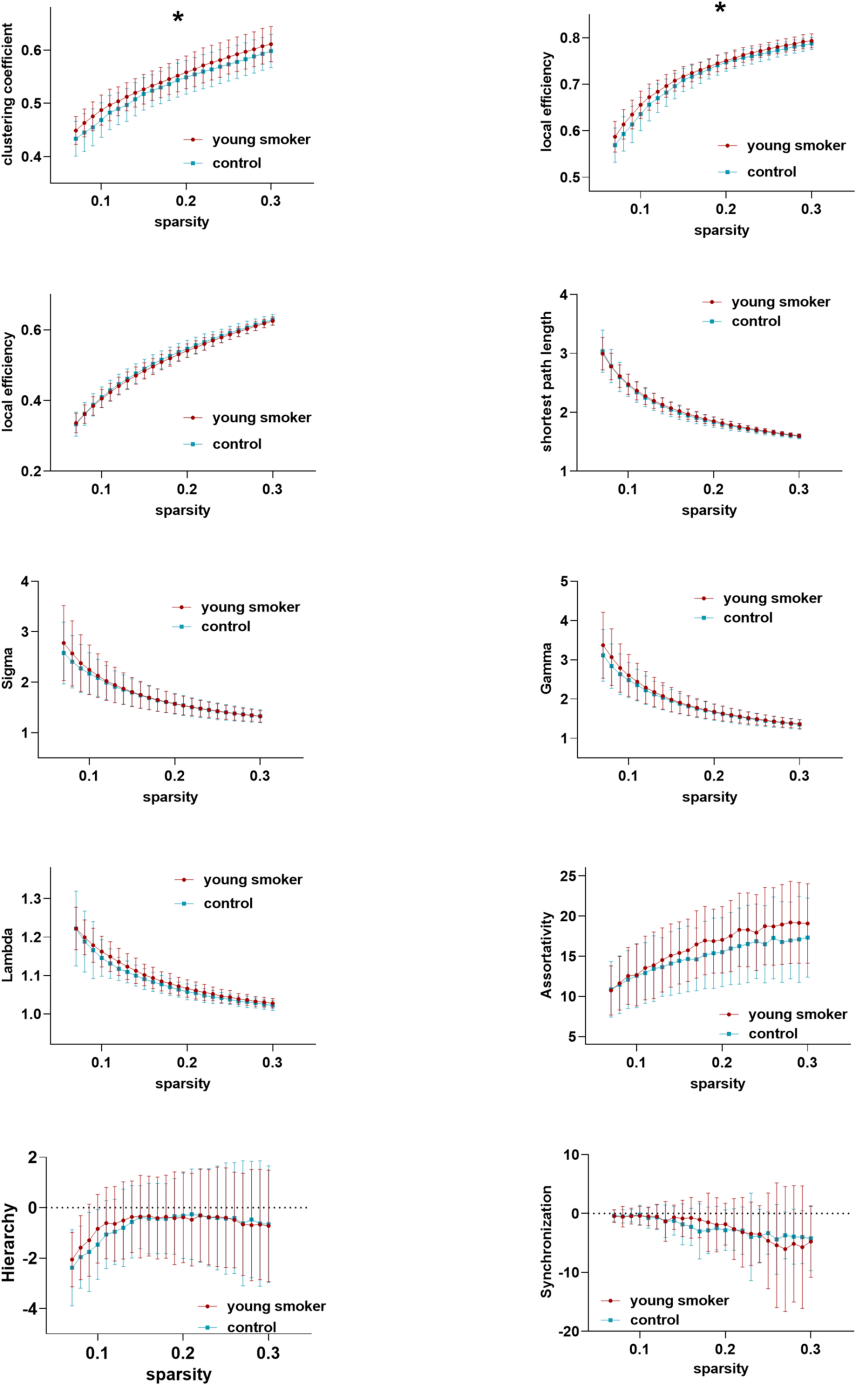
Quantification of small‐world topological properties of WM functional networks in young smokers and healthy controls. Coloured error bars indicate the SD of topology properties across sparsities.

Throughout the entire range of sparsity in the WM functional connectome, all participants in the young smokers and healthy controls (HCs) showed σ values higher than 1.1 (the WM correlation network of both groups demonstrated a small‐world property). Compared to HCs, the young smokers group demonstrated a significantly higher $C_{p}$ (*t* = 2.155, *p* = 0.034*), and $E_{\text{local}}$ (*t* = 2.419, *p* = 0.017*) on AUC; however, there was no significant difference between the young smokers and HCs regarding λ (*t* = 0.090, *p* = 0.090), σ (*t* = 0.639, *p* = 0.639), γ (*t* = 0.414, *p* = 0.414), $L_{p}$ (*t* = 0.484, *p* = 0.484), $E_{\text{global}}$ (*t* = −1.014, *p* = 0.313), β (*t* = 0.316, *p* = 0.752), α (*t* = 0.133, *p* = 0.133), and S (*t* = −0.069, *p* = 0.944) on AUC (Table 2, Figure 2 and Figure 3). Bonferroni correction was used for the 10 planned comparisons.

**TABLE 2:** Group differences of global properties.

	$C_{p}$	$E_{\text{local}}$	$L_{p}$	$E_{\text{global}}$		
**P**	0.034^a^	0.017^a^	0.484	0.313		
**T**	2.155	2.419	0.702	−1.014		
	**λ**	**γ**	**σ**	**α**	**β**	**S**
**P**	0.090	0.414	0.639	0.133	0.752	0.944
**T**	1.713	0.819	0.470	1.513	0.316	−0.069

^a^ Significant alterations.

### 3.3 Group Differences of Nodal Properties

**FIGURE 4:**
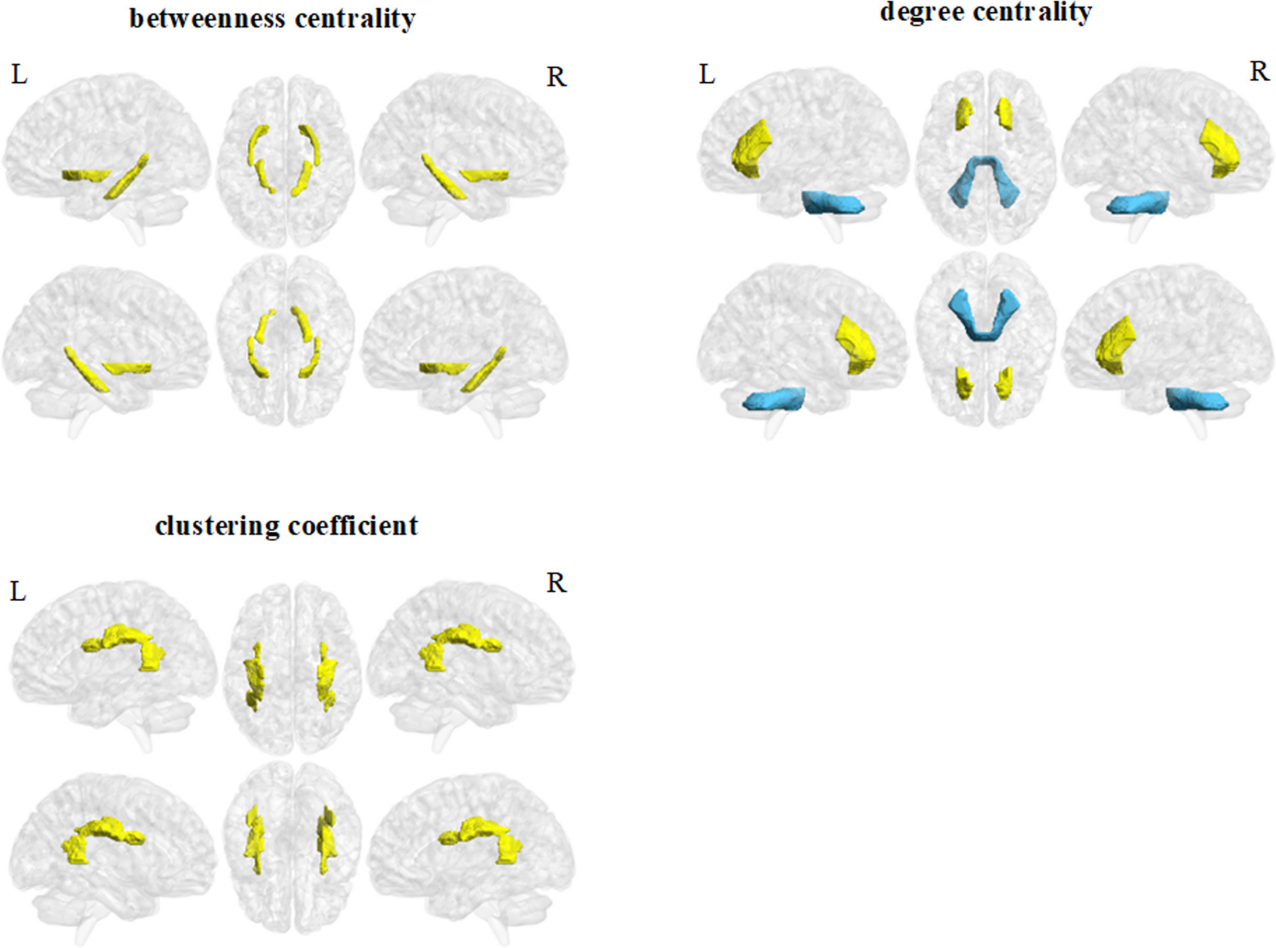
Group differences of nodal properties between the young smokers and HCs. The yellow regions represent higher and blue regions represent lower nodal betweenness centrality, nodal degree centrality or nodal clustering coefficient in the young smokers compared with HCs.

In contrast to the HCs group, the young smokers group showed decreased node degree centrality in node 61 (located in the middle cerebellar peduncle) and increased node degree centrality in node 72 (located in the bilateral anterior radiation crown); increased node betweenness centrality was also observed in node 4 (located in the Cingulum (hippocampus)) and node 84 (located in the inferior fronto‐occipital fasciculus) of smokers. Moreover, the young smokers group demonstrated increased node clustering coefficient in node 48 (located in the superior longitudinal fasciculus) compared to the HCs group. There were no significant differences between the HCs group and the young smokers group in terms of node efficiency, local node efficiency, and shortest path length of nodes. The statistical results were corrected using the Bonferroni correction (Table 3 and Figure 4).

**TABLE 3:** Group differences of nodal properties.

Nodal order	Localization in JHU‐Atlas	*p*	T
**Betweenness centrality**			
4	Cingulum (hippocampus)	0.0001^a^	3.902
84	Inferior fronto‐occipital fasciculus	0.0002^a^	3.859
**Degree centrality**			
61	Middle cerebellar peduncle	0.0001^a^	−3.946
72	Anterior corona radiata	0.0001^a^	3.991
**Clustering coefficient**			
48	Superior longitudinal fasciculus	0.0003^a^	3.730

^a^ *p* < 0.05, Bonferroni correction.

### 3.4 Clinical Relevance Analysis

**FIGURE 5:**
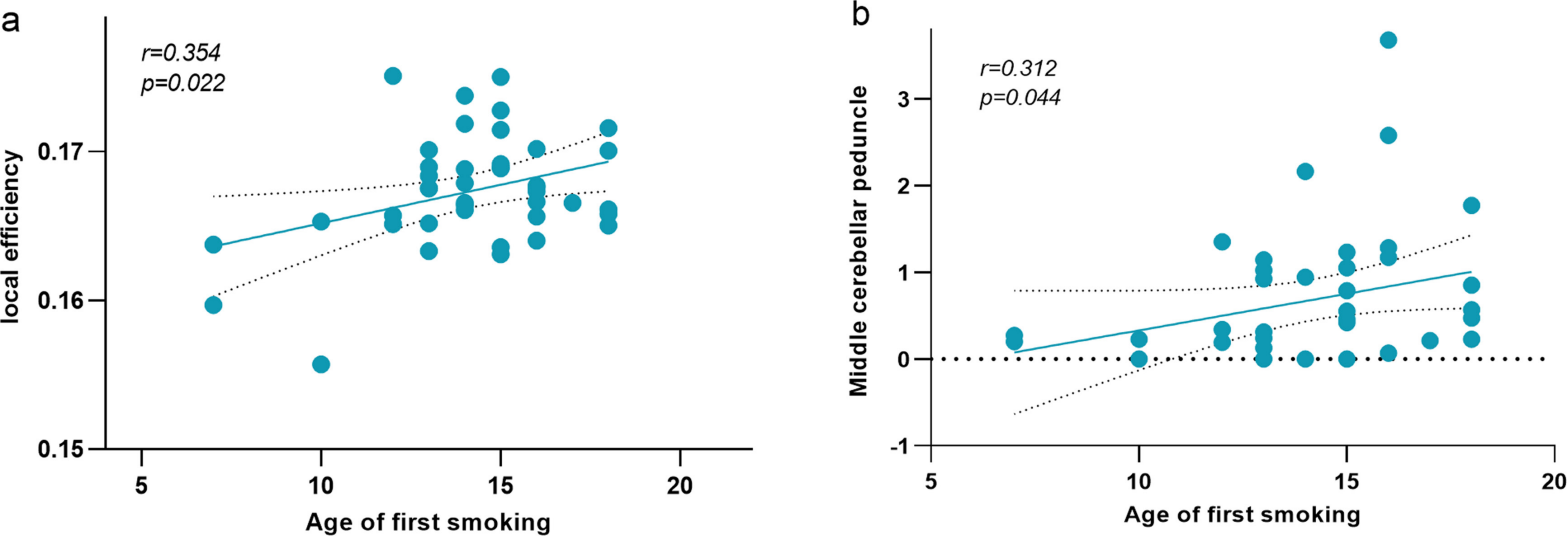
Clinical relevance analysis (a, b). The young smokers group showed a positive correlation between higher $E_{\text{local}}$ and age of first smoking (a). The node degree centrality value of node 61 was positively correlated with age of first smoking (b).

Pearson correlation analysis showed that, in contrast to the HCs group, the young smokers group showed a positive correlation between higher $E_{\text{local}}$ and age of first smoking (*r* = 0.354, *p* = 0.022, Bonferroni corrected) (Figure 5a). Spearman correlation analysis showed that the node degree centrality value of node 61 was positively correlated with age of first smoking (*r* = 0.312, *p* = 0.044, Bonferroni corrected) (Figure 5b).

## 4 Discussion

This study utilized graph theory analysis to investigate the topological properties of the WM functional network in young smokers. Results showed that both the young smokers and non‐smokers exhibited small‐world characteristics in the WM functional connectomes. In terms of global topological properties, compared with the control group, the smoking group showed increased $C_{p}$ and $E_{\text{local}}$; and the increased $E_{\text{local}}$ was positively correlated with the age of first smoking. In terms of node topological properties, young smokers exhibited abnormal node properties in WM regions of the Cingulum (hippocampus) (CGH), bilateral superior longitudinal fasciculus (SLF), bilateral inferior fronto‐occipital fasciculus (IFO), middle cerebellar peduncle (MCP), and the bilateral anterior corona radiata (ACR). The node degree centrality value of MCP was positively correlated with age of first smoking. These findings may promote our understanding of the disruption of young smokers WM function caused by nicotine addiction from a functional topological perspective and provide further evidence for the neural mechanisms underlying nicotine addiction.

Complex network analysis has been to be a new multidisciplinary approach to studying brain functions, aiming to characterize topological properties of brain networks that are both neurobiologically meaningful and computationally feasible [41]. It has been demonstrated that the human brain is organized in a “small‐world” pattern, which is both high‐value and low‐energy‐cost [41]; this could mean that it balances global integration and local segregation capabilities during information processing, exhibiting high levels of both global and local efficiency [42, 43, 44].

At the global level, in this study, both the young smokers group and the control group exhibited small‐world characteristics in the WM functional connectome, with all participants' σ values exceeding 1.1. The present results indicated that the global and local information can be processed in parallel, consistent with previous research on the small‐world structure of the WM functional connectome [40].$E_{\text{local}}$ measures the efficiency of information transmission within the neighbourhood of each node in the network, reflecting the fault tolerance and information redundancy of local subnetworks [40]. In the current study, the $E_{\text{local}}$ in the young smokers was significant increased, which indicates stronger local information processing capacity, suggesting that information transfer within cliquishness is faster in young smokers [45]. $C_{p}$ is a metric that measures the degree of interconnection between nodes and their neighbouring nodes in a network [40]. In small‐world topological properties, $C_{p}$ reflect network segregation, network segregation is positively correlated with $C_{p}$[42, 44, 46]. Network segregation refers to the probability of communication between a selected brain region and other brain regions within small‐scale local brain networks [40, 47]. In our result, the young smokers showed decreased elevated $C_{p}$ and $E_{\text{local}}$, which implied that the young smokers showed a non‐optimized structure of WM network in both global and local level properties. Our result supports previous studies using diffusion tensor imaging (DTI) to investigate changes in the topological organization of the WM structural network in young smokers, namely increased $E_{\text{global}}$ and $E_{\text{local}}$ [30], further supporting previous research that long‐term smoking may lead to widespread structural and functional brain damage [2]. This study is in line with former findings on chronic smokers [48, 49]. Compared with the nonsmokers, chronic smokers showed higher $E_{\text{local}}$ and $C_{p}$ [48, 49], which indicated that WM function topology is of great significance to help us understand the pathogenesis of nicotine addiction and may contribute to the development of interventions for nicotine addiction [48, 49].

At the node level, young smokers exhibited abnormal node properties in WM regions of the Cingulum (hippocampus) (CGH), bilateral superior longitudinal fasciculus (SLF), bilateral inferior fronto‐occipital fasciculus (IFO), middle cerebellar peduncle (MCP), and the bilateral anterior corona radiata (ACR). These abnormal WM regions overlapped with regions found in previous studies focused on white matter structural abnormalities and fibre tract integrity. For example, compared with non‐smokers, young smokers showed increased fractional anisotropy (FA) in the bilateral superior longitudinal fasciculus, cingulate, left anterior corona, left superior corona, left posterior corona, and left frontoparietal tract [10, 50, 51]; and young smokers also exhibited higher FA in the nearly symmetrical bilateral frontopaietal bundles (comprising the primary WM pathway SLF) compared to non‐smokers [52]. Subsequent studies showed that the significant increase in FA was observed in the corpus callosum (CC), bilateral internal capsule (IC), and portions of the left SLF in young smokers [51]. $NC_{p}$ measures the probability of a node connecting to its neighbouring nodes, reflecting isolation function, indicating the probability of communication between selected brain regions and other brain regions within small‐scale local networks [40, 47]. Abnormal $NC_{p}$ in the young smokers group suggests altered information transmission capacity in local brain regions, impaired local information integration and segregation functions. Abnormal $C_{p}$ and $NC_{p}$ in the young smokers group, possibly due to changes in the topological properties of certain critical nodes affecting the topological properties of the entire brain network. In general, each node in a network is a fundamental unit of the system, and the efficiency of neural information integration and the stability of network structures in the brain are significantly influenced by the nodes [53]. Changes in the topological properties of certain key nodes in the brain may affect the topological properties of the entire brain network. The current results found that these regions include the CGH, SLF, IFO, MCP and ACR, which may be key regions in the WM functional network, providing potential targets for future research on adolescent smoking addiction.

## 5 Limitation

This study has certain limitations that must be acknowledged. First, our results are based on a relatively small sample size and should therefore be considered preliminary. Further studies should include more participants and may even be divided into different subtypes. Additionally, this study is cross‐sectional in design and cannot confirm the causal relationship between WM topological structure changes and smoking severity. Longitudinal studies should be conducted in the future to assess these effects. Third, in this preliminary study, we did not have diffusion MRI to further support our findings.

## Author Contributions

**Zhenzhen Mai:** writing – original draft, validation, methodology, formal analysis, data curation, conceptualization. **Dahua Yu:** writing – review and editing, funding acquisition, formal analysis, data curation, conceptualization. **Gengdi Huang:** writing – review and editing, funding acquisition. **Xiaojiao Li:** methodology, formal analysis. **Xuwen Wang:** resources, investigation. **Fang Dong:** data curation, visualization. **Yongxin Cheng:** formal analysis. **Juan Wang:** software. **Yuxin Ma:** resources. **Lin Luo:** writing – review and editing. **Kai Yuan:** writing – review and editing. **Ting Xue:** writing – review and editing, funding acquisition, conceptualization.

## Funding

This work was supported by Chinese National Programs for Brain Science and Brain‐like Intelligence Technology (2022ZD0214500); the STI2030‐Major Project (2022ZD0207100); the National Natural Science Foundation of China (82260359, 82371500, U22A20303, 61971451); the Natural Science Foundation of Inner Mongolia (2023QN08007, 2025MS08027, 2025MS08098); the Fundamental Research Funds for the Universities of Inner Mongolia (2023QNJS204, 2023QNJS206, 2024QNJS119); the Development Program for Young Talents of Science and Technology in Universities of Inner Mongolia (NJYT24030); and the Project of the Inner Mongolia Talent Program (Yingcai Xingmeng) Brain Science and Brain‐Like Computing Team.

## Ethics Statement

This study received approval from the Medical Ethics Committee of the First Affiliated Hospital of Baotou Medical College of Inner Mongolia University of Science and Technology, and complied with the Declaration of Helsinki. The study had been registered with the Chinese Clinical Trial Registry (No. ChiCTR2100042449). All participants and their legal guardians signed written informed consent forms after fully understanding the purpose of the study.

## Conflicts of Interest

The authors declare no conflicts of interest.

## Data Availability

The datasets presented in this article are not readily available because of the privacy of all the participants. Requests to access the datasets should be directed to the corresponding authors.
